# A handheld flexible manipulator system for frontal sinus surgery

**DOI:** 10.1007/s11548-020-02220-0

**Published:** 2020-07-01

**Authors:** Suat Coemert, Robert Roth, Gero Strauss, Pia M. Schmitz, Tim C. Lueth

**Affiliations:** 1 Institute of Micro Technology and Medical Device Technology, Technical University of Munich, Boltzmannstr. 15, 85748 Garching, Germany; 2 IRDC GmbH International Reference and Development Centre for Surgical Technology, Käthe-Kollwitz-Straße 64, 04109 Leipzig, Germany

**Keywords:** Flexible manipulator, Frontal sinus surgery, Handheld, Endoscopic surgery

## Abstract

**Purpose:**

Draf drainage is the standard treatment procedure for frontal sinus diseases. In this procedure, rigid angled endoscopes and rigid curved instruments are used. However, laterally located pathologies in the frontal sinus cannot be reached with rigid instrumentation. In order to assist surgeons with such complicated cases, we propose a novel handheld flexible manipulator system.

**Methods:**

A cross section of 3 mm × 4.6 mm enables transnasal guiding of a flexible endoscope with 1.4 mm diameter and a standard flexible surgical instrument with up to 1.8 mm diameter into the frontal sinus with increased reachability. The developed system consists of an electrical discharge-machined flexure hinge-based nitinol manipulator arm and a purely mechanical handheld control unit. The corresponding control unit enables upward and left–right bending of the manipulator arm, translation, rolling, actuation and also quick exchange of the surgical instrument. In order to verify the fulfillment of performance requirements, tests regarding reachability and payload capacity were conducted.

**Results:**

Reachability tests showed that the manipulator arm can be inserted into the frontal sinus and reach its lateral regions following a Draf IIa procedure. The system can exert forces of at least 2 N in the vertical direction and 1 N in the lateral direction which is sufficient for manipulation of frontal sinus pathologies.

**Conclusion:**

Considering the fact that the anatomical requirements of the frontal sinus are not addressed satisfactorily in the development of prospective flexible instruments, the proposed system shows great potential in terms of therapeutic use owing to its small cross section and dexterity.

**Electronic supplementary material:**

The online version of this article (10.1007/s11548-020-02220-0) contains supplementary material, which is available to authorized users.

## Introduction

### Clinical background

**Fig. 1:**
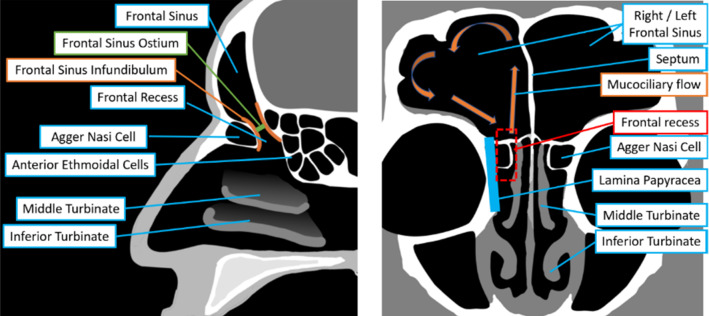
Frontal sinus anatomy: saggital view (left), coronal view (right)

The frontal sinus is one of the most variable human anatomical structures. Its size and shape is highly dependent on race and climate [1, 2]. The proportions and shape of the frontal sinus and neighboring anatomical structures can sometimes vary substantially. The frontal sinus (Fig. 1) usually consists of two air-filled volumes situated behind the right and left part of the forehead. They are separated in the left and the right frontal sinus by the frontal septum in the sagittal plane. In adults, both frontal sinuses are roughly pyramid shaped. In the frontal sinus floor, there is a conically tapering constriction, the frontal sinus infundibulum [3]. It leads to the smallest constriction of the passage to the frontal sinus, the frontal ostium. It is the opening of the frontal sinus to the natural frontal sinus outflow tract (frontal recess). The frontal sinus outflow tract leads downward around the agger nasi cell into the nasal cavity. From here, there exists a path which leads past the middle turbinate to the nostril and the outside air. In normal mucociliary flow, the secretions are transported laterally over the superior mucosa, and move back over the inferior mucosa before finally draining into the frontal recess [4].

Of frontal sinus diseases frontal rhinosinusitis is the most common [1]. Frontal rhinosinusitis can be initiated by many different causes such as bacterial infection, fungal infection and tumors, such as mucoceles and inverted papillomas. Symptoms for frontal rhinosinusitis are facial pain, headache, blockage of the nose and reduced or lacking sense of smell. Furthermore, frontal sinus tumors can compress surrounding structures, sometimes resulting in sight loss.

### State of the art

The state-of-the-art treatment method of frontal sinus diseases is the endoscopic Draf drainage [5]. In this procedure, a rigid endoscope and a rigid surgical instrument need to be guided through the given anatomical constrictions in order to reach the frontal sinus from the nose. Anatomical variability in the number of frontal cells is common and can change the accessibility of the frontal sinus. By cutting and drilling from a transnasal approach, a hole can be opened and some of the internal bone structures are removed to assist the drainage of accumulating mucus, which facilitates mucociliary flow in the frontal sinus. Also, some pathological tissues can be reached directly through the wider opening of the frontal sinus floor. The Draf classification system for transnasal surgery of the frontal sinus consists of three basic categories, Draf I, Draf II and Draf III, where accessibility increases with invasivity, respectively. In a type Draf I procedure, only a small hole is drilled in the frontal sinus floor and some anterior nasal cells are removed. Due to the small diameter of the opening this is primarily useful for drainage. In contrast, in a Draf type II procedure, more of the anterior nasal cells are removed to increase maneuvering space for endoscopes and instrumentation. The agger nasi cell and the frontal sinus floor are removed up to the lateral orbit to permit access to the frontal sinus. A distinction is made between type IIa drainage and type IIb drainage. Type IIa drainage is achieved by resection of the bony frontal sinus floor on one side between the lamina papyracea and the middle turbinate of the nose. In type IIb drainage, the bony floor of the sinus cavity is removed with a diamond drill beyond the head of the middle nasal concha to the septum. For Draf III, the same structures are removed on both sides, while the nasal septum between both areas is removed. Despite the higher invasiveness of the method, Draf III grants the best accessibility for transnasal treatment of difficult areas in the frontal sinus and is still preferred to methods with external approach.

In open surgery, the frontal sinus is accessed from the outside by operating through a hole or osteoplastic flap that is opened in the patient’s forehead [1]. Although access to pathological tissues is excellent in this method, the high invasivity of the procedure encompasses a significantly higher risk of complications. Often endoscopic access is tried prior to open approaches. External surgery is used in cases where the endoscopic approach fails, especially when treating benign and malignant frontal sinus pathology lateral to the lamina papyracea [6]. Frontal sinus trephination is used to reach mucoceles and small osteomas in lateral regions of the frontal sinus, while osteoplastic flaps are used for treatment of cerebrospinal fluid leaks [1].

Endoscopic frontal sinus approaches are performed with rigid endoscopes and instruments. For endoscopic view of lateral regions of the frontal sinus, angled endoscopes ranging from 30° up to 70° are commonly used [7]. Here, the optics enables a field of view twice larger than that of the human eye [8]. Also, several types of handheld rigid instruments exist, which are applicable for frontal sinus surgery. Similar to the endoscopes, the rigid instruments are also available at many different angles to enable greater treatment coverage for the diverse locations of pathology.

### Limitations of state-of-the-art

Recent advances in the field of transnasal surgery have significantly augmented the capabilities of surgical treatment of the sinuses. Nevertheless, access to superior and lateral regions of the frontal sinuses proves to be problematic with existing surgical instruments [1, 6]. Due to complex anatomical configuration, the surgical passage to the frontal sinus remains difficult. The more distant pathological tissue is situated from central regions, the higher the degree of invasiveness that is required for surgical access. In such cases, open surgery becomes inevitable [9]. Occasionally, even external approaches may not suffice on their own [1]. Furthermore, external procedures such as frontal sinus trephination and osteoplastic flaps have disadvantages including risk of nerve damage (supraorbital nerve), scarring and high blood loss. Therefore, in cases when open surgery is believed to be necessary for a successful treatment of pathology, often the acceptance of the pathology is chosen over the risk presented by surgery.

### State of research

In research, several approaches have been developed to address the limited maneuverability of the state-of-the-art systems. To allow operating in anatomically confined workspaces, steerable surgical instruments should be minimized in diameter and should be flexible with distributed bending. Still, to enable controlled treatment of pathologies, sufficient manipulation forces need to be supplied with the appropriate types of tools and visualization. A transnasal robotic system proposed by Wirz et al. for transsphenoidal skull base surgery, which was tested in cadaveric experiments, uses pre-curved concentric tubes, which facilitate inflected extension by the translation of the internal tubes. [10] However, such concentric tube robots need to allow considerable elastic bending of the inner tubes to permit guiding through outer tubes of different curvature, which reduces the achievable manipulation forces [11]. For endoscopic endonasal surgery, Gerboni et al. presents the bioinspired HelixFlex system, which is multi-actuatable using axial and helical actuation cables [12]. The outer diameter of the most recent prototype is 5.8 mm, which is reasonably close to a size which might allow access to the frontal sinus. However, the instrument channel is only 1 mm in diameter which limits capabilities of pathology manipulation. Yoon et al. proposed a robotic system which consists of separate endoscope and biopsy end effector modules with 4 mm and 5 mm diameters, respectively [13]. Due to size limitations, the simultaneous insertion of both modules is not feasible in the frontal sinus area.

Handheld instruments are explicitly desired in ENT surgery, since the surgeons prefer having maximized control of instruments by using their hands, as motivated by their practical training. The primary challenge is to extend the dexterity of the existing tools, but at the same time, to provide the corresponding functions within a compact handheld unit in an intuitive way. Awtar et al. presented a handheld flexible surgical tool (FlexDex) which translates the wrist motions of the surgeon into the end effector’s motions (bending in two planes and rolling) [14]. This device targets an ergonomic and intuitive control of the end effector by its parallel kinematic based frame, which is attached to the surgeon’s arm and connected to a pull-wire transmission mechanism. The surgical system Canady Robotics SR-70 (*US Medical Innovations, Takoma Park, USA*), previously known as JAIMY, is a similar handheld articulating laparascopic instrument with 5 mm diameter which offers 3 degrees of freedom (roll, pitch, yaw). This robotic system originates from the work of Zahraee et al. and distinguishes itself from the FlexDex system with the motorized actuation of the instrument functions [15]. Another intuitive approach to transfer the control inputs into the end effector motion is the bioinspired multi-actuation method implemented in HelixFlex [12]. The actuation method, which is inspired by squid tentacles, is based on simultaneous actuation of one parallel and two helically oriented cables, which results in four degrees of freedom of the multi-maneuverable tip.

### Novel approach

In order that the aforementioned complicated cases are treated, the risk of surgical procedure needs to be reduced. Here, it stands to reason that improved instrumentation enabling sufficient transnasal access to superior and lateral regions of the frontal sinus, would significantly reduce risks posed by treatment. With this purpose in mind, we develop a flexible system to assist surgeons with the complicated cases. Thanks to its flexibility, the system should be able to reach areas in the frontal sinus than cannot be reached easily with rigid instruments. Besides the flexibility, the main novelty of the developed device is enabling maximized spaces for a surgical tool (2 mm instrument channel) and an endoscope (1.6 mm endoscope channel) within a very small cross section (3 mm × 4.6 mm). Despite having a similar size to articulating laparoscopic instruments with comparable degrees of freedom (such as the mentioned FlexDex or SR-70 systems), the developed system allows both visualization and manipulation functions within this scale. Moreover, while the presented robotic solutions for transnasal surgery in the literature cannot address the size limitations of frontal sinus anatomy satisfactorily, the proposed system distinguishes itself with its small cross section which increases its chances in terms of potential therapeutic use in frontal sinus surgery. The most decisive effort to realize these features is to combine the design and manufacturing process in an effective way. These efforts will be elaborated in the following section.

## Realization

### From anatomical measurements to design requirements

**Fig. 2:**
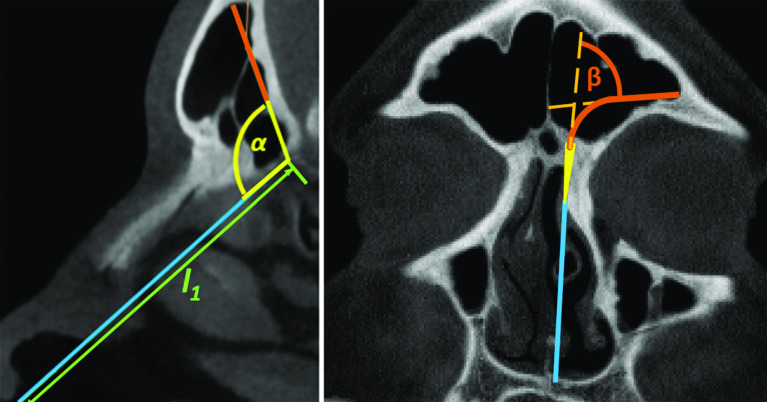
Anatomical measurements of the angles and the lengths for accessing superior and lateral regions of the frontal sinus through the nostrils

For a reliable manipulator design, a good understanding of frontal sinus anatomy and its access routes is essential. With this purpose, manual measurements were performed on sagittal frontal sinus views of up to 30 anonymous patients. The length $l_{1}$ and the angle $\alpha$ were measured on those views (Fig. 2 left). From the same data set, coronal views of the 10 laterally widest frontal sinuses (with at least 35 mm width) were selected to measure the second characteristic angle $\beta$ (Fig. 2 right).

One of the most important parameters for the manipulator design is the length $l_{1}$ which helps estimating the minimum required length of the rigid segment of the manipulator. The mean value for $l_{1}$ was found to be 59.6 ± 4.3 mm. Another decisive measure for the design is the angle $\alpha$, which indicates the required bending angle between insertion axis through the nostril and the entrance axis into the frontal sinus to reach the superior regions of frontal sinus. The mean value for the angle 180° − $\alpha$ was found to be 69.1° ± 11.2°. Measurements of the angle $\beta$ revealed that bending angles around 75° would be sufficient to reach the lateral regions of frontal sinuses for the majority of patients. For the four patients where a part of the lateral outline would be unreachable in the theoretical measurement, this area never spanned more than 5 mm height in total. Nevertheless, in practice, such areas could likely be reached by simple opening of the forceps instrument.

### Design

**Fig. 3:**
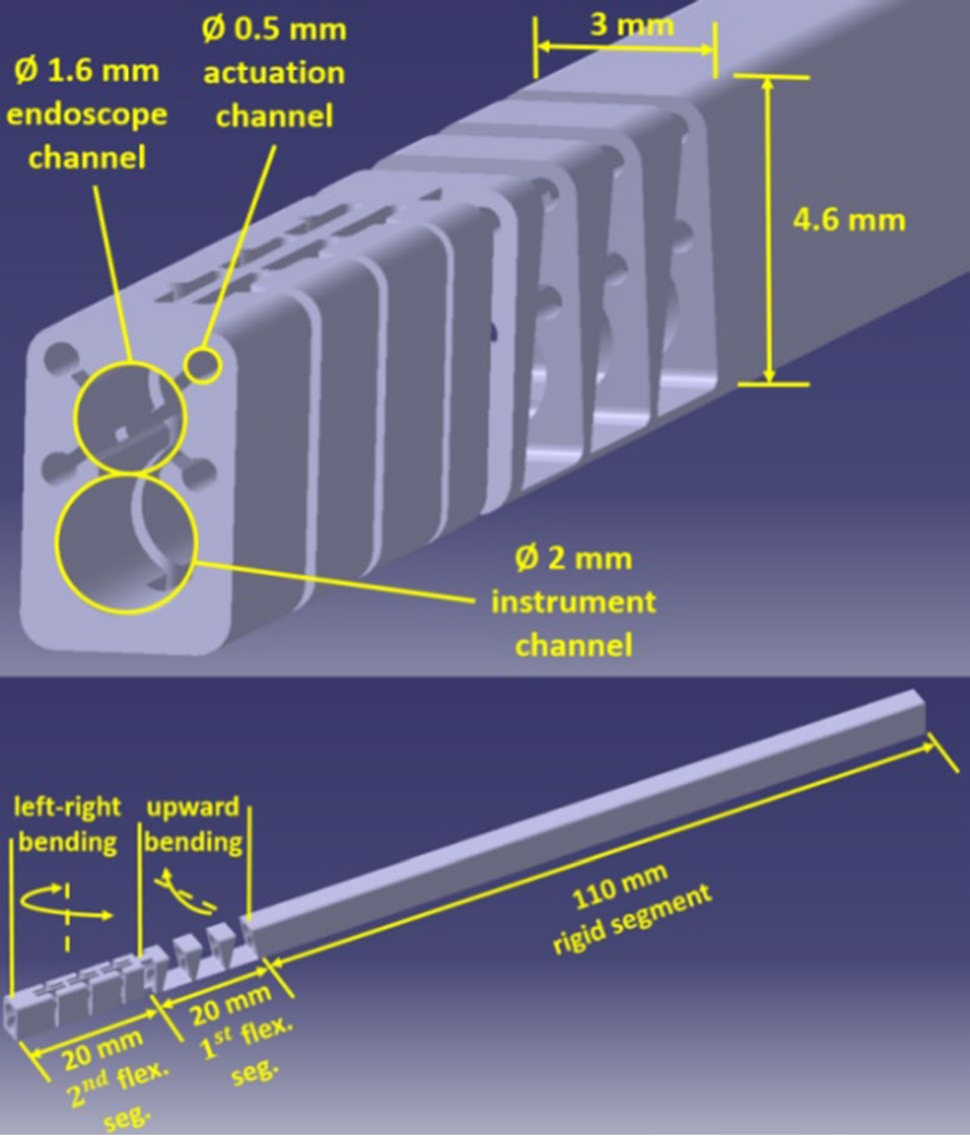
Manipulator design

*Manipulator arm design* To be able to reach first the superior region and second the lateral region of the frontal sinus, respectively, two independently bendable flexible segments are needed. The first segment is supposed to provide a smooth insertion into the frontal sinus by overcoming the angle (180° − $\alpha$) to reach the superior regions. The second segment is intended for extension of the device to the lateral regions of the sinus. The design incorporates the use of a flexible endoscope for visualization and a flexible instrument for therapy. Nevertheless, it should be also kept in mind that the size needs to be minimized with the purpose of limiting the invasiveness of the Draf drainage procedure to Type IIa. The hole in the frontal sinus floor which is made during a Draf IIa procedure is found to have an average minimum diameter of 5.6 mm in the literature [16]. To be able to pass the manipulator through this opening, a cross section of 3 mm × 4.6 mm and 150 mm length was determined as manipulator dimensions (Fig. 3). The largest diameter (diagonal) of the manipulator cross section is around 5.1 mm and smaller than the aforementioned diameter required for Draf IIa. The 150 mm length was determined considering the conducted anatomic measurements ($l_{1}$ in Fig. 2, max. width and height of representative large frontal sinuses) and the length of standard rigid frontal sinus instruments. Within the cross section, a 1.6 mm channel for the use of a flexible endoscope and a 2-mm channel for the use of a flexible surgical instrument are provided. As a flexible videoscope, minnieScope-XS (*Enable Inc., Redwood City, USA*) with 1.4 mm diameter, integrated illumination and 1 Megapixel resolution can be utilized to inspect the frontal sinus satisfactorily. As flexible instrument a grasping forceps with 1.8 mm diameter is conceivable for manipulation tasks. After positioning the manipulator within the frontal sinus in a desired orientation, the system can be mounted on an OR table clamp. In this way, the surgeon can translate the endoscope relative to the surgical tool by one hand for an optimal visualization of the tool. The selected clearance between the channel diameter and the tool diameter allows translation and rotation of the endoscope by the user but also prevents its unintentional movements by means of sufficient friction in the channel. The other hand can be used for the actuation of the tool.

For the actuation of flexible segments, 4 channels with 0.5 mm diameter are needed, the two upper channels for the first and the two lower channels for the second flexible segment. Through its 150 mm length, the manipulator consists of one rigid segment of 110 mm and two flexible segments of 20 mm. The first flexible segment is responsible for the bending to enter the frontal sinus with an optimal orientation. According to the anatomical measurements for the angle (180° − α), the first flexible segment was designed in a way that it can provide up to 75°. In this way, the superior region can be reached by turning around the corner between the frontal sinus opening and nasal cavity, where ethmoidal cells are located that are standardly removed during the Draf drainage procedure. The second flexible segment is responsible for providing the bending to reach the lateral regions of the frontal sinus. Here, also a bending up to 75° is expected to be sufficient to reach the regions that cannot easily be reached with rigid optics and instruments.

Flexible segments are realized with flexure hinges that are favorable for a compact design and elimination of the assembly [17, 18]. This type of design is very advantageous for simplifying the miniaturization and sterilizability, however the deflection provided by flexure hinges is limited. Therefore, the use of a highly elastic material is necessary. To address this limitation, nitinol is a good choice due to its superelasticity. Moreover, it has a good biocompatibility and is suitable for creating thin-walled structures thanks to its high strength, which is another favorable aspect for miniaturization. Since realization of such a challenging design using such an expensive material is associated with high costs, reusability of the manipulator must be ensured to make it a cost efficient product, especially by taking sterilizability aspects into account.

**Fig. 4:**
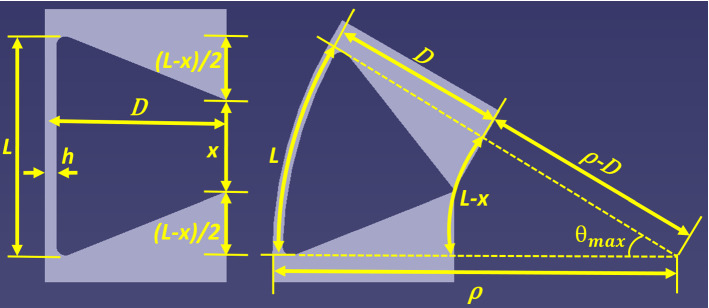
Design of mechanical stops for constraining the bending angle

Recently, we have conducted an experimental work to determine the maximum elastic bending angle of nitinol flexure hinges with rectangular cross section [19]. In this work, a simple design guideline based on flexure hinge length *L* and thickness *h* (both dimensions shown in Fig. 4) was proposed to calculate the maximum elastic bending angle $\theta_{max}$ in degrees:

1 $$\theta _{{\max }} \left[ {\deg } \right] = \frac{{3L}}{{2h}}$$

In the same work, the consistency of the proposed guideline was verified using FEM simulations. It was shown that deflection angles proposed in the guideline yield to operating strains between 4.5% and 4.7%, which result in load cycles between 10,000 and 100,000 for nitinol [20].

If we consider that flexible segments should consist of multiple serial flexure hinges for a continuum movement, an uneven stress distribution can be foreseen due to varying lever arms in case of distal loading, as long as each hinge is of identical geometry. Therefore, it is sensible to stiffen the hinges furthest from the distal end and soften the closest ones in order to get a uniform stress and strain distribution. Although this is a very evident and essential measure for continuum compliant structures, it is only marginally implemented in the literature. This results in reduced performance of continuum compliant structures in terms of compliance, payload capacity and durability.

Using the guideline in Eq. (1) and in light of optimization considerations in the previous paragraph, both flexible segments were each designed with three flexure hinges of 5 mm length and thicknesses of 0.35 mm, 0.30 mm and 0.25 mm beginning from the proximal one to the distal one, respectively. The selection on the number of flexure hinges allows some flexibility, since rather the total length is decisive to achieve a certain maximum elastic deflection angle. On the other hand, the guideline in Eq. (1) was determined for hinge lengths between 3 mm and 8 mm. Therefore, 2 to 5 elements are conceivable. However, having too many elements requiring many rigid sections in between would result in an increased radius of curvature. Having only two elements might lead to sharp bending in the curved anatomy and the mentioned optimization concept is less effective in terms of the curvature and stress distribution. Consequently, 3 hinges with 5 mm length were found to be the most ideal solution. Flexure hinges in each segment can elastically bend maximum up to 21°, 25° and 30°, respectively, which result in a total of 76° within the 16 mm length including the 0.5-mm-long rigid portions in between the flexure hinges which corresponds to a bending radius of approximately 12 mm. Angular stops were designed to limit the bending angle to avoid plastic deformation. While flexure hinges do not bend with constant curvature, the gap width *x*, which functions as a mechanical stop, was determined using this geometrical relationship assuming that the inaccuracies will not result in major differences. In Fig. 4, this geometrical relationship is also illustrated. If a flexure hinge with a length of *L* bends up to the mechanical stop with a maximum angle $\theta_{\hbox{max} }$ and a bending radius *ρ*, the gap width *x* will be dependent on *D* (the distance between the neutral axis of the flexure hinge and the gap) and $\theta_{\hbox{max} }$. This relationship is derived in Eqs. 2, 3, 4 and 5.

2 $$ L = \rho  \theta_{{\max}} $$

3 $$ L - x = \left( {\rho - D} \right) \theta_{{\max }} $$

4 $$ \rho  \theta_{{\max }} - x = \rho  \theta_{{\max }} - D \theta_{{\max }} $$

5 $$ x = D \theta_{{\max }} $$

**Fig. 5:**
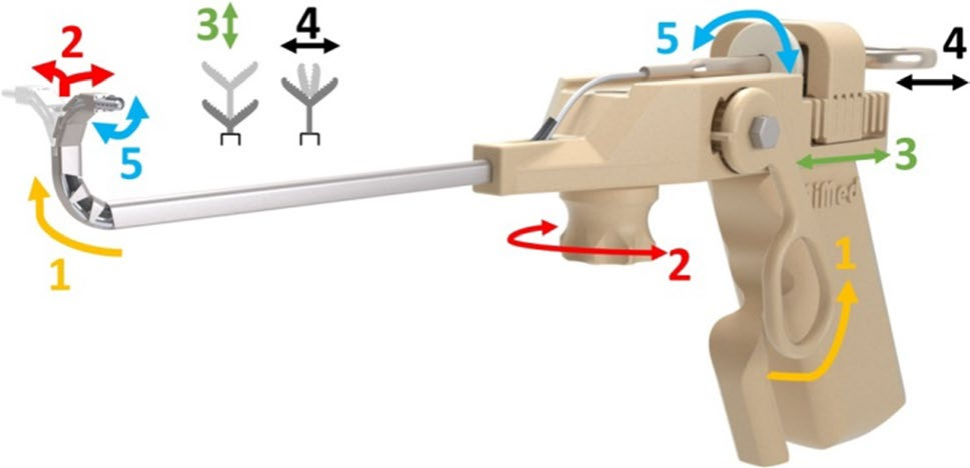
Operating principle of the handheld control unit

*Handheld control unit design* To enable the features of the manipulator, the realization of a handheld control unit is required. The operating principle is illustrated in Fig. 5. The designed control unit enables upward motion of the first flexible segment (1) and left–right motion of the second flexible segment (2). It also facilitates back-and-forth translation (3), actuation (4) and rolling (5) of the surgical instrument. With this pistol-like design, three of these features can be operated by just the right hand, (1) with the middle finger, (2) with the index finger and (4) with the thumb. Translation (3) and rolling (5) of the instrument can be provided with the help of the left hand.

Motion transmission from control levers to the flexible segments is provided by flexible stainless steel wire ropes with 0.35 mm diameter and minimum breaking force of 135 N. Wire ropes are fixed at the manipulator tip and also at the shafts attached to the control levers. Back-and-forth translation of the ropes is enabled by rotation of the cylindrical shafts that are inside the hubs dedicated to them within the main block. The shaft orientations are chosen to correspond directly to the respective tip motions in endoscopic view. The left–right control is more distal than the up-down control to correspond intuitively to the bending segments which have the same order.

In order to maintain the manipulator pose even if the operating surgeon removes his fingers from the levers, a self-locking feature is implemented by choosing shaft-hub tolerances that generate sufficient friction.

**Fig. 6:**
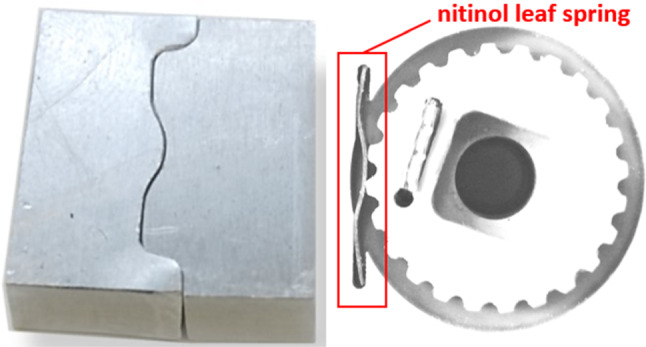
Two-part mold milled for shape setting (left), through shape setting manufactured nitinol leaf spring (right)

Considering the usability risk that operating with friction-based control levers might lead to user fatigue by static friction effects, an alternative self-locking feature was implemented using custom-made nitinol leaf springs. In the implementation stage of this concept, two challenges needed to be tackled. First, there exists no standard leaf spring in the desired shape and size to purchase. Secondly, the leaf spring should exhibit high amount of elastic deflections in order to fulfill its function. At this point, nitinol provides convenience with its two unique properties to deal with these challenges: superelasticity and shape setting ability. For the manufacturing of nitinol leaf springs, superelastic nitinol sheets with 0.25 mm thickness (*Euroflex GmbH, Pforzheim, Germany*) were first cut into the desired widths and lengths. Subsequently, the sheets were constrained inside the two-part aluminum mold milled with the desired contour. Lastly, the shape setting was performed at 500 °C temperature for 20 min and followed by a water quench. Figure 6 shows the shape setting mold and the manufactured leaf spring.

### Manufacturing

*Manufacturing of the Manipulator Arm* The suitability of electrical discharge machining (EDM) for realizing such challenging designs like the manipulator had been reported in an earlier work [21]. Advantages worth mentioning are the ability of machining small features (i.e., actuation channels with 0.5 mm diameter and wall thicknesses as small as 0.2 mm) through long workpieces (i.e., length up to 150 mm), accurate production of flexure hinges (i.e., max. ± 5% variation in the thickness of 0.3 mm). Considering these advantages, the manipulator arm design in Fig. 3 was realized with EDM.

As a disadvantage of the EDM process, the contamination of the brass wire on the surface of the machined parts can be mentioned. For manufacturing medical devices, this issue can be critical regarding toxicity, and therefore, appropriate measures must be considered. To deal with the contamination issue caused by brass wire during EDM, the applicability of a chemical etching process was investigated in a previous work of our group [22]. Here, cytotoxicity testing was conducted in accordance to the current standard DIN EN ISO 10993-5 using Human fibroblast cells (type Hs27). Here, the cleaned samples showed no cytotoxicity. This process was also implemented for the manipulator arm.

**Fig. 7:**
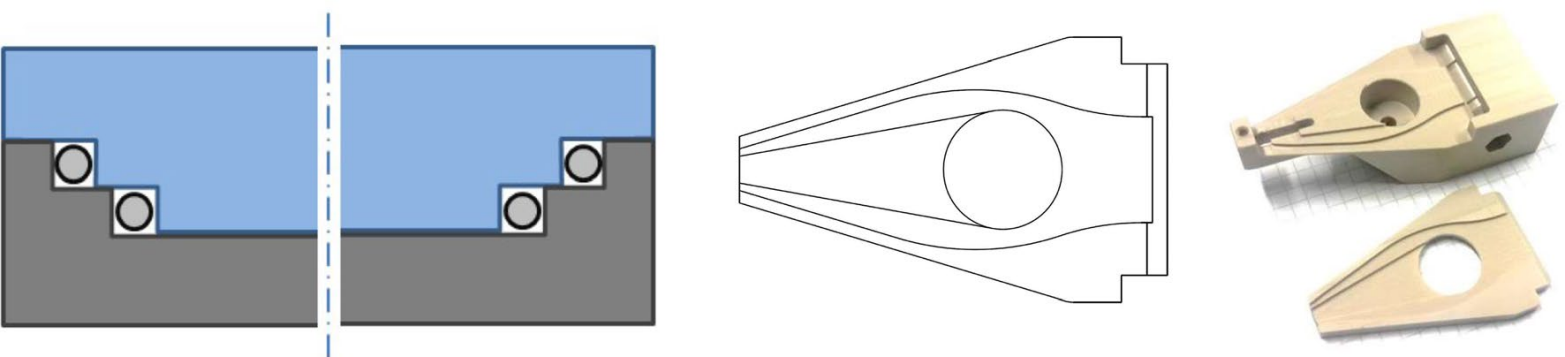
Manufacturing concept of the actuation guides: abstracted cross section of two part design, which forms rectangular actuation guides (left), top view of lower part showing the curve of the four cable guides (middle), manufactured cable guide parts before assembly (right)

*Manufacturing of the Control Unit* For the control unit, we followed a conventional manufacturing approach by milling and turning of PEEK, considering its biocompatible, mechanically robust nature and easy processability. A crucial feature of the control unit is the transmission of tension forces from the levers to the manipulator tip. Therefore, narrow actuation guides need to be fabricated to facilitate the Bowden cable principle. The developed manufacturing solution for this is characterized by its two-part design. Here, the main body and the add-on part form rectangular wire guides by the horizontal offset between the stepped parts as shown in Fig. 7 (left). This concept is applied along the desired curved cable path Fig. 7 (middle). The two manufactured parts (Fig. 7, right) were assembled using the medical device adhesive Loctite EAM-121HP (Henkel Adhesives, Dusseldorf, Germany).

**Fig. 8:**
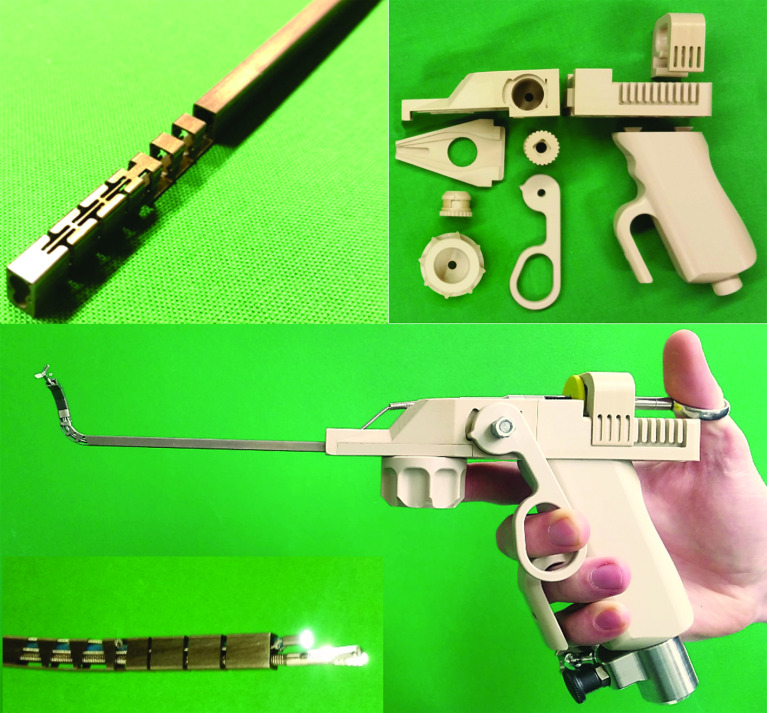
The manufactured manipulator arm (top left) and the components of the control unit before the assembly (top right) and the complete manipulator system after the assembly with closeup of manipulator tip (bottom)

Subsequent to manufacturing all components, the system consisting of the manipulator arm and the control unit was assembled. Figure 8 shows the manufactured components before the assembly and the complete system after the assembly, as well as the hand position during control with ergonomic stabilization by the support bow on the tool handle.

## Verification

### Reachability test

**Fig. 9:**
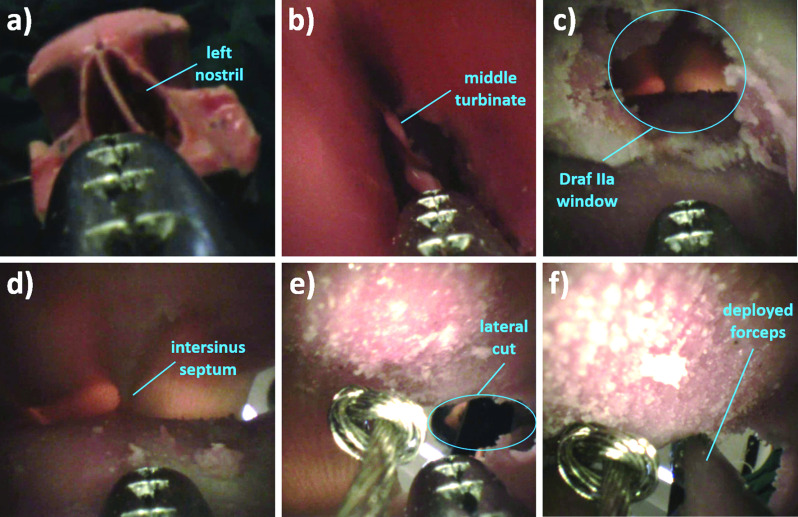
Image seqence **a** before insertion through the left nostril **b** upon insertion through the left nostril **c** after first bending upward, before entering the frontal sinus through the Draf IIa window **d** upon entering the frontal sinus **e** after second bending to the right **f** upon deployment of the forceps to the lateral region of the sinus

**Fig. 10:**
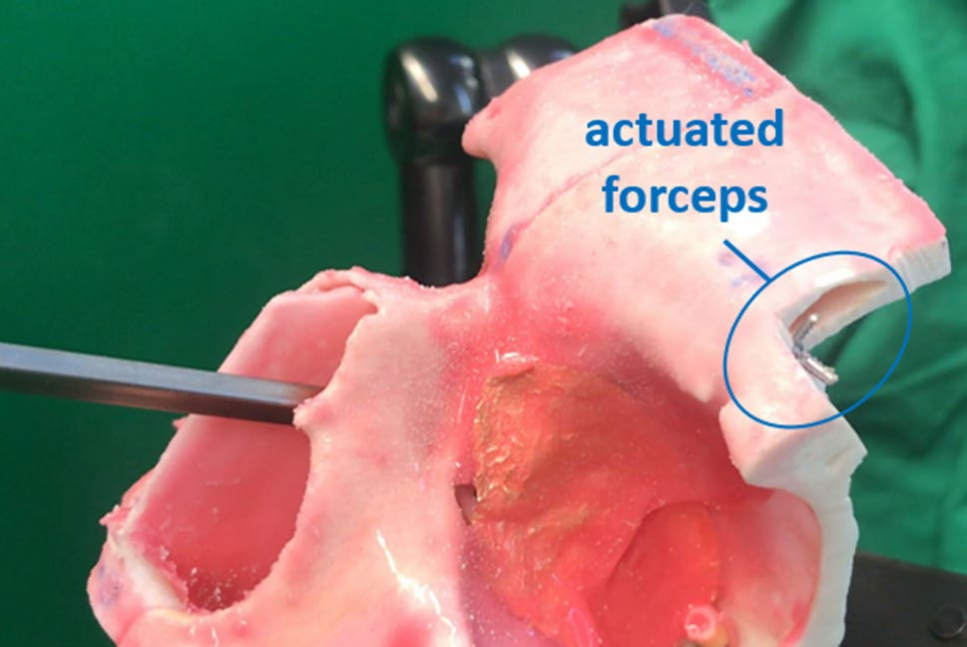
Actuation of the end effector upon deployment at the lateral region of the frontal sinus

Lateral reachability of the manipulator system was verified using a commercially available frontal sinus phantom (*model SN*-*ab sinus patient “Meyer”, PHACON GmbH, Leipzig, Germany*) which is used for training of surgeons for functional endoscopic sinus surgery (FESS). The phantom has a regular frontal sinus anatomy and realistically represents the anatomy of a 40-year-old patient. Upon request, a Draf IIa procedure was performed on the left frontal sinus of the phantom by an experienced surgeon at the International Reference and Development Center for Surgical Technology (*IRDC GmbH, Leipzig, Germany*). Using the operated phantom, the procedure to reach lateral regions of the sinus was simulated with the developed system under endoscopic view. Here, the aforementioned flexible endoscope minnieScope-XS was used. The lateral part of the sinus was cut open to be able to observe the manipulator tip from outside. After several practice trials, the user (with no clinical experience) was able to enter the frontal sinus, reach the lateral region and retract the system within a minute. Figure 9 shows sequences indicating some important landmarks from the video captured by the endoscope and Fig. 10 shows the moment at which the end effector is actuated upon reaching the lateral region.

### Payload measurements

**Fig. 11:**
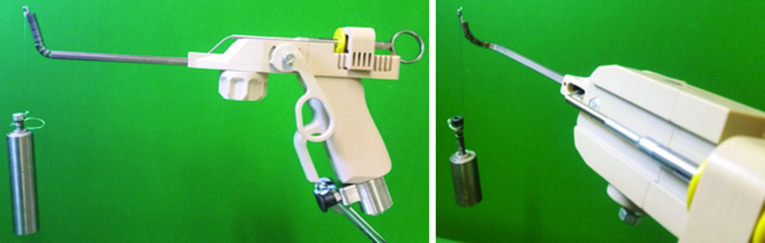
Payload measurements of the manipulator: loading cases against the upward bending of the first segment (left) and against the right bending of the second segment (left)

Beside the reachability, payload capacity was another concern for the manipulator design. The manipulation of pathological tissues necessitates transmitting sufficient forces from the control unit to the manipulator tip. Payload measurements were conducted for two loading cases. In the first case, loads were applied in the vertical downward direction against the upward bending of the first flexible segment (Fig. 11 left). In the second case, the manipulator system was tilted in a way that loads could be applied in the vertical downward direction against the right bending of the second flexible segment (Fig. 11 right). Due to high manufacturing costs, the manipulator was not risked to see the limits of the payload capacity. Nevertheless, it could be observed that the system was able to withstand 2 N in the first loading case and 1 N in the second loading case without any failure. Bekeny et al. reported that required forces during in vitro and in vivo excision of pituitary tumors were in the range of 0.1–0.5 N [23]. Based on this information, we can claim that the manipulator is able to exert sufficient forces for interacting with pathological structures in the frontal sinus.

## Discussion

As a preliminary step toward validating efficacy and usability, the developed system was evaluated by Prof. Dr. Gero Strauss on a frontal sinus phantom similar to the one used in the reachability test. The evaluation was documented by means of an evaluation questionnaire which included questions regarding potential advantages of the system in terms of reaching hard-to-reach pathologies, decreasing postoperative trauma and the manipulators functions, the shape-locking feature, intuitiveness and ergonomics. Prof. Strauss confirmed that lateral pathologies can be reached more easily with the developed system compared to the conventional instruments and invasiveness of the procedure can be reduced. He also confirmed the benefits of the manipulator functions and shape-locking feature. Moreover, he confirmed that the system can be operated intuitively and ergonomically. Additionally, fixation of the system on an adjustable OR table clamp (Fig. 11 left) after positioning the manipulator at the pathological site was perceived positively, since otherwise operating with a floating system in the hand for a longer period would result in user fatigue. In this configuration, more delicate actions can be performed.

To reduce user fatigue and to increase repeatability, a robotic version of the developed tool would be conceivable. Here, the loss of haptics could prove problematic, especially for the prevention of excessive manipulator forces against resistance, which would therefore also necessitate a good three dimensional tracking over anatomical images and assisted path planning, which might sometimes be difficult due to high anatomical variation and soft tissue displacement in the approach to the frontal sinus.

In terms of risk management, the durability of flexure hinges is of great importance. It must be ensured that flexural elements have sufficient fatigue life to eliminate any possibility of malfunction during the procedure which could lead to critical consequences. Therefore, an FEM-based optimization tool was developed for flexure hinge-based continuum structures [24]. The developed algorithm automatically analyzes the stress distribution under certain loading and deformation conditions of initial designs defined by the user, adjusts a uniform stress distribution among individual flexure hinges by automated dimensioning and finalizes the design by integrating mechanical stops automatically matched with the kinematic capacity of individual hinges. In this work, flexure hinge-based structures similar to the flexible segments of the frontal sinus manipulator were subjected to fatigue tests, and the effect of the optimization method on the fatigue life was investigated. The results confirmed that the fatigue life during pulsating angular displacement can be increased up to 100% through the optimization and fatigue cycles in the order of 40,000–70,000 can be achieved. These values indicate that the system would be able to present sufficient durability for a repeated use.

Another potential limitation of the device would be the channel size allocated for the surgical tool which might present a challenge in terms of manipulation forces when interacting with larger pathologies. This challenge could be handled by developing task-specific novel tools using smart materials such as nitinol which are able to expand upon deployment (i.e., nitinol basket). Alternatively, the size of the cross section can be increased in order to allocate a larger channel for the surgical tool. In this case, however, a more invasive procedure such as Draf III would be needed to still allow frontal sinus access.

Lastly, the high costs can be mentioned as a limitation of the developed system, which originate from high processing times related to conventional machining methods such as wire EDM and CNC machining. Given the fact that the system is intended as a reusable device, its manufacturing cost in the order of several thousand dollars might still be profitable in case of a commercialization. Future advances in additive manufacturing technology might significantly help reducing these costs, by allowing rapid manufacturing of complex geometries. However, to allow this, some of the current limitations stated in [21] should be resolved appropriately, especially those limitations which are related to the production of compliant miniaturized snake-like structures.

## Conclusion

In this work, we proposed a novel flexible handheld manipulator system for particular frontal sinus operations where standard rigid endoscopes and instruments are insufficient in terms of reachability. In the design procedure, the frontal sinus anatomy was analyzed to determine the design requirements. Thanks to the monolithic design specifically adapted to the anatomy, manufacturing and material capabilities, a miniaturized dexterous system could be realized which, despite its small cross section, allows the use of a flexible videoscope and a surgical tool. To verify the expected advantages of the developed system performance tests were performed. The conducted phantom test revealed that the realized system can reach the lateral region of a regular size frontal sinus following a Draf IIa procedure, as targeted in the designing process, whereas with rigid instruments lateral pathologies cannot be reached even in more invasive Draf III cases. Furthermore, the payload capacity over 1 N indicates that the system can generate sufficient forces for interactions with pathological tissues in the frontal sinus. In the future, the clinical safety of the system should be confirmed by certified testing laboratories. Finally, the ultimate aim is the evaluation of the system by clinical experts in the real clinical context to validate the clinical efficacy.

## Electronic supplementary material

Below is the link to the electronic supplementary material.

Supplementary material 1 (AVI 35625 kb)
